# A modified aeroponic system for growing small-seeded legumes and other plants to study root systems

**DOI:** 10.1186/s13007-023-01000-6

**Published:** 2023-03-03

**Authors:** Jingya Cai, Vijaykumar Veerappan, Kate Arildsen, Catrina Sullivan, Megan Piechowicz, Julia Frugoli, Rebecca Dickstein

**Affiliations:** 1 Department of Biological Sciences and BioDiscovery Institute, University of North Texas, Denton, TX 76203 USA; 2 Department of Biology, Eastern Connecticut State University, Willimantic, CT 06226 USA; 3 Department of Genetics and Biochemistry, Clemson University, Clemson, SC 29634 USA

**Keywords:** Aeroponic system, Legumes, Symbiotic nitrogen fixation, *Medicago truncatula*, Root systems

## Abstract

**Background:**

Various growth systems are available for studying plant root growth and plant–microbe interactions including hydroponics and aeroponics. Although some of these systems work well with *Arabidopsis thaliana* and smaller cereal model plants, they may not scale up as well for use with hundreds of plants at a time from a larger plant species. The aim of this study is to present step-by-step instructions for fabricating an aeroponic system, also called a “caisson,” that has been in use in several legume research labs studying the development of symbiotic nitrogen fixing nodules, but for which detailed directions are not currently available. The aeroponic system is reusable and is adaptable for many other types of investigations besides root nodulation.

**Results:**

An aeroponic system that is affordable and reusable was adapted from a design invented by French engineer René Odorico. It consists of two main components: a modified trash can with a lid of holes and a commercially available industrial humidifier that is waterproofed with silicon sealant. The humidifier generates a mist in which plant roots grow, suspended from holes in trash can lid. Results from use of the aeroponic system have been available in the scientific community for decades; it has a record as a workhorse in the lab.

**Conclusions:**

Aeroponic systems present a convenient way for researchers to grow plants for studying root systems and plant–microbe interactions in root systems. They are particularly attractive for phenotyping roots and following the progress of nodule development in legumes. Advantages include the ability to precisely control the growth medium in which the plants grow and easy observations of roots during growth. In this system, mechanical shear potentially killing microbes found in some other types of aeroponic devices is not an issue. Disadvantages of aeroponic systems include the likelihood of altered root physiology compared to root growth on soil and other solid substrates and the need to have separate aeroponic systems for comparing plant responses to different microbial strains.

**Supplementary Information:**

The online version contains supplementary material available at 10.1186/s13007-023-01000-6.

## Background

Soil-less culture is a method to grow plants by using nutrient solution without soil, either by hydroponic systems [1, 2] or aeroponic systems [3]. Different methods have been devised for hydroponics, with specific applications dependent on the plant species, growth conditions, budget etc. In hydroponic setups, plant roots are typically immersed in a nutrient solution with the rest of the plant supported above the solution or roots may be subjected to nutrient solutions pumped to them. In other hydroponic systems, roots may be subjected to a nutrient solution stream flowing in a piped system, or roots may be subjected to periodic immersion in a nutrient solution or plant roots may be part of an aquaponics setup in which nutrients are recycled from a fish tank (reviewed in [2]). Aeroponics can be thought of as an extension of hydroponics, one in which the nutrient solution is aerosolized and the plant roots grow in the resulting mist with the rest of the plant suspended above [3]. Both hydroponic and aeroponic systems can provide a controlled growth environment for plant research: both facilitate growing plants in regulated nutrient conditions and allow the researcher to easily alter the nutrient conditions as desired. In genetic studies, especially for root phenotypes, both aeroponic or hydroponic systems can also reduce the labor and allow high-throughput of screening mutants’ phenotypes. After screening in an aeroponic or hydroponic system, selected plants may be transferred to soil for further growth and analysis.

Soil-less systems are particularly useful for studying nitrogen-fixing nodule development in legume roots and more than several labs, including ours, have reported nodulation studies using aeroponic or hydroponic system devices [4–12]; see Additional file 1: File S1 for additional studies that have used aeroponic systems. The system that many of us use and is described here is based on the design originated by René Odorico in the Laboratoire des Interactions Plantes-Microrganismes, Toulouse, France (LIPM) [4] and subsequently modified by Douglas Cook of the University of California, Davis to accommodate up to 800 plants. This aeroponic system is also called a “caisson,” French for hermetic box. We have further adapted it to use more easily available components. Using this system, it is easy to observe a root nodule development time course, although our modifications of the design no longer incorporate the window into the root area of the original aeroponic system [4]. Previous protocols for aeroponic system devices similar to ours have been made available via the internet [4], but these web pages have subsequently gone defunct.

**Fig. 1:**
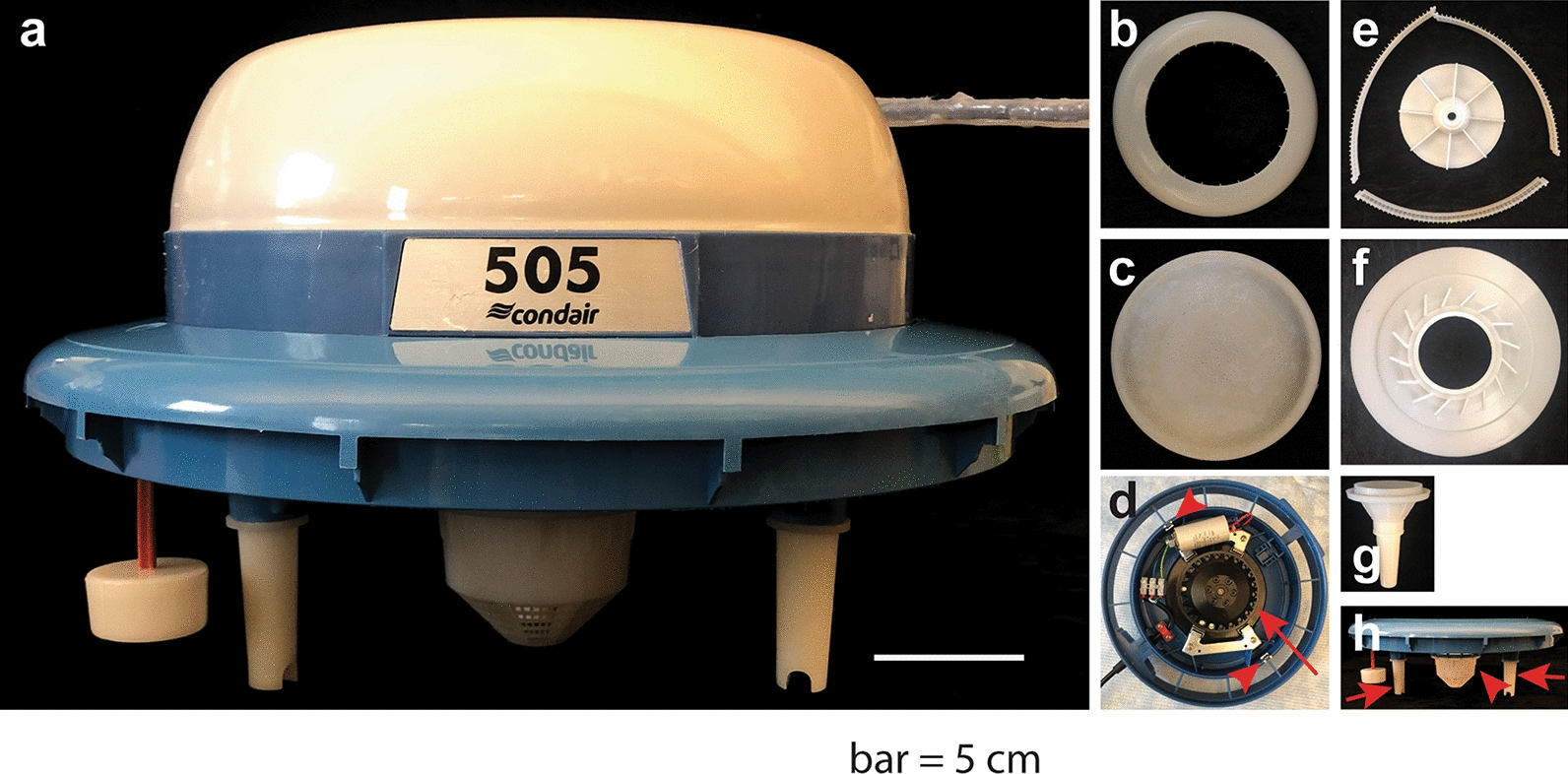
Parts in the aeroponic system “motor.” **a** The Condair 505 humidifier used in the aeroponic system is composed of the parts shown in panels **b**–**h** Bar = 5 cm. **b** It contains a plastic aerosol discharge hood; **c** a rounded plate protecting the inner electric and motorized parts affixed to the base by two screws and **d** base on which the electric and motorized parts are mounted. The red arrow points to the rotary motor and the red arrowheads point to the metal screw guides. **e** The guiding disk and parts for the atomizer ring fit onto the underside of the base as does a whirl disk (**f**); and a suction piece (**g**). **h** The humidifier support base fused with the white integrated filter (red arrowhead). The three base stand pegs are removable for cleaning (red arrows show two that are visible in the image). Not shown is a white plastic reservoir that fits underneath the base that is part of the humidifier but which is not used in the aeroponic system

As noted above, aeroponic systems take the basic ideas of hydroponic systems [1–3] a step further with plants’ roots suspended in a mist rather than a solution [3, 4]. An advantage of aeroponic systems over hydroponic systems is that aeroponic systems provide both humidification to the roots, while also improving the roots’ exposure to oxygen. Most aeroponic systems aerosolize the nutrient solution by using an ultrasonic atomization fogger or a pressure atomization nozzle [2]. An advantage to using these types of atomizers is that they can create very fine particles of nutrient solution in mist form. The disadvantage for plant–microbe interactions is that they can shear microbes that are placed in the medium, so that the researcher may lose active microbes. In this report, we describe the fabrication of an alternative device: a mechanical humidifier that creates a mist with larger particles created by spinning the nutrient solution through various parts that aerosolize the medium (Fig. 1).

## Results and discussion

### Aeroponic system “motor” and preparation for use

The heart of an aeroponic system is a reliable misting/fogging device. Unlike most systems that employ atomizer nozzles with small orifices to create a mist, such as pressure atomization nozzles or ultrasonic foggers [3], our system relies on a mechanical spinning disk to create a mist. The advantage for using this system in legume nodulation or other plant root-microbe interactions studies is that the mister has low shear forces and does not mechanically disrupt the microbes, which may be an issue with other systems. The Condair 505 humidifier (Condair Ltd, Pfäffikon, Switzerland) [13, 14], a rotational atomizer, fills this role in our system. Our groups have found this humidifier to be well suited for use in an aeroponic system and easily modified for this use; other humidifiers that carry out a similar function may also work.

The humidifier (Fig. 1a) is composed of the parts shown in Fig. 1b–h. It contains a plastic aerosol discharge hood (Fig. 1b) that fits over the rounded plate (Fig. 1c) and snaps into the assembled base; a rounded plate that protects the inner electric and motorized parts (Fig. 1c); and a base on which the electric and motorized parts are mounted, in the center of which is the rotary motor (red arrow) (Fig. 1d). Two metal screw guides (Fig. 1d, red arrowheads) allow the rounded plate (Fig. 1c) to be affixed to the base. The underside of the humidifier contains a guiding disk and parts for the atomizer ring (Fig. 1e), for which slots exist on the underside of the base; a whirl disk (Fig. 1f); and a suction piece (Fig. 1g). When assembled, the humidifier sits on a support base with an integrated filter (Fig. 1h, red arrowhead) and three stand pegs (Fig. 1h, red arrows; the third stand peg is behind the filter in the image) into which the humidifier nests. The humidifier is sold with a white plastic reservoir, which is not used in the aeroponic system.

**Fig. 2:**
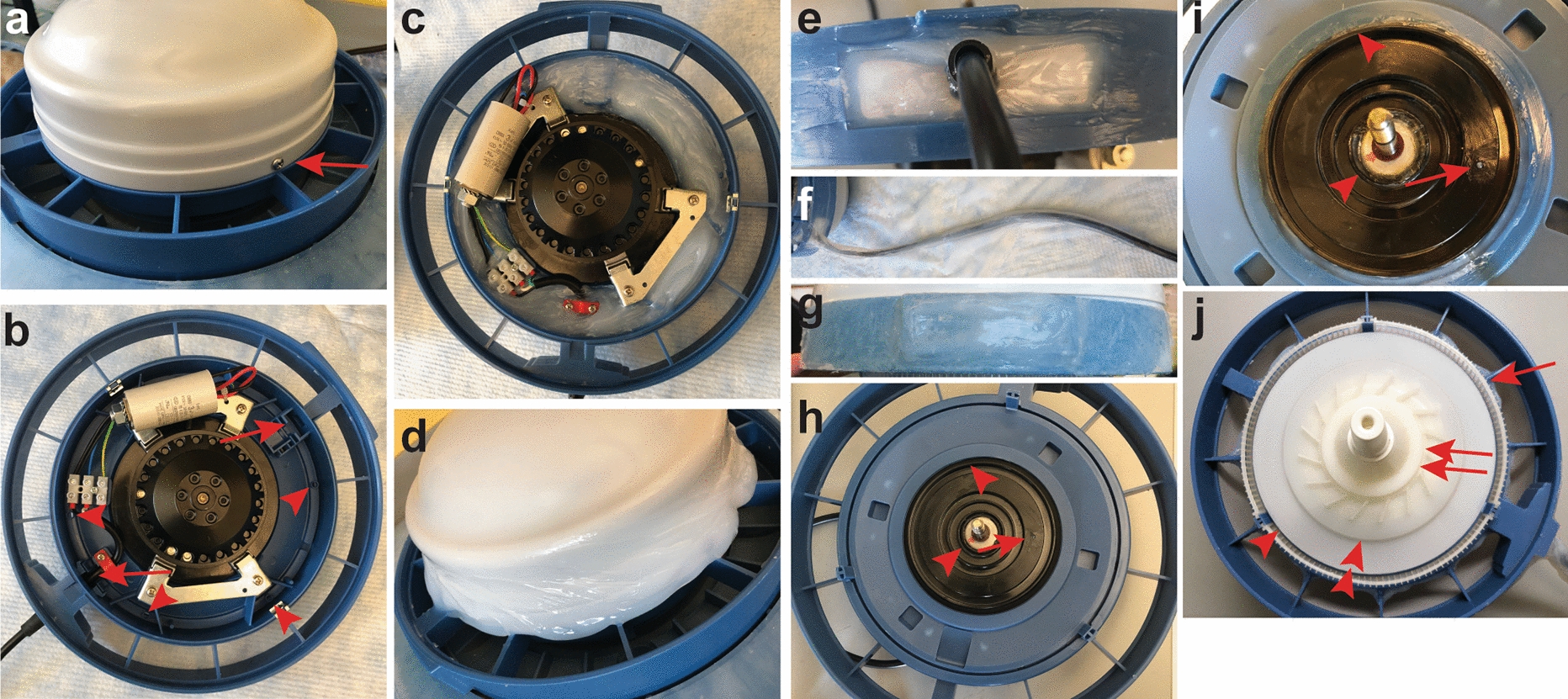
Waterproofing the aeroponic system “motor.” **a** The humidifier rounded plate is anchored to the base by two screws, one of which is visible (red arrow). Unscrewing these opens the humidifier. **b** Once the rounded plate is removed, the six holes that must be sealed (red arrowheads; two are behind the capacitor), the electric cord entry and another hole (red arrows) into the electric parts that must be sealed are visible. **c** The interior of the base is filled with silicone sealant. **d** After the metal plate is reattached to the base with its screws, the seam is sealed. **e** The entry point for electric power to enter the base is sealed. **f** The electric cord is sealed. **g** The opposite side of the base from the power entry site, where there is an opening is sealed. **h** The underside of the base. The hole in the black disk motor mount is visible (red arrow). **i** The underside of the base after sealing. The hole in the black disk motor mount is sealed (red arrow). A thin bead of sealant is applied between the black disk motor mount and the other parts of the base (red arrowheads). **j** Parts are reassembled onto the motor shaft in order: whirl disk (single arrowhead), guiding disk (double arrowheads) with the suction piece (double arrows) screwed onto the motor shaft firmly to hold the pieces in place. The three-piece atomizer ring parts (single arrow) should then be slotted into place on the bottom of the support disk

Before use, sensitive parts of the humidifier are sealed with silicone sealant to prevent water-based solutions from entering the inside of the humidifier or other electric parts. In order to accomplish this, the discharge hood (Fig. 1b) and rounded plate (Fig. 1c) need to be removed from the humidifier base (Fig. 1d). The rounded plate is removed by unscrewing the two screws on either side of it that hold it in place (Fig. 2a). The removable plastic parts on the underside of the humidifier base (Fig. 1e–g) should also be disassembled from the base. Then a sealant should be applied to all open orifices. Although the color of the sealant is not consequential, it is important to use sealant that does not contain fungicides or other agents that will interfere with or harm experimental organisms. For our experiments, we use aquarium-safe sealant (ASI Aquarium Sealant; Best Materials, Phoenix, AZ). Waterproofing the humidifier is, at minimum, a two-day process to allow the sealant to completely dry and “cure,” but usually is a three- to four-day process.

On day one, the base should be sealed. There are six holes in the base where the atomizer ring fits into the base (arrowheads in Fig. 2b; only four are visible in the image with the other two hidden behind the capacitor and wiring to the capacitor) and gaps where the electric cord and supports enter the base (Fig. 2b, arrows). These should be filled with sealant. In practice, it is easier to fill the base with sealant (Fig. 2c), taking care to avoid having the sealant come into contact with the metal parts affixed to the base because the acid in the sealant can corrode the metal. The sealant in the base should then be allowed to dry/cure for 24–48 h. After the sealant is dry, the second step is to assemble the rounded plate onto the base using the two metal screw guides (Figs. 1d, 2a arrow) with the screws provided by the humidifier manufacturer. Subsequently, the silicone sealant should be applied to seal the gap between the rounded metal plate and the humidifier base (Fig. 2d shows a liberal application of sealant). The place where the electric cord enters the base should be sealed at the same time (Fig. 2e); we continue the sealant up the cord close to where it sits at the top of the modified trash bin to prevent small droplets of water sliding down the flexible cord (Fig. 2f). The opposite side of the humidifier from the place where the electric cord enters the base has a nameplate and an opening to the base. It should be sealed at the same time (Fig. 2g, note that in this humidifier the nameplate has been removed). These should be left to dry/cure for 24–48 h.

The next step is to seal the underside of the humidifier base. In principle, this could be done at the same time that the inside of the base is sealed, but we find it easier to wait until after the rounded plate (Fig. 1c) is re-assembled onto the base, silicon-sealed and dried/cured. To waterproof the underside of the base, turn the partially assembled humidifier upside down to apply sealant to the underside. This allows gravity to help keep the sealant in place until it dries. On the underside, gaps around the black disk that is mounted to the base and around the motor shaft should be sealed (Fig. 2g, i, arrowheads). Figure 2h shows the underside before sealing and Fig. 2i shows the underside after sealing. There is also hole in the black disk motor mount that should be sealed (Fig. 2g, i, arrow). It is vital not to apply too much sealant to the area around the motor shaft so that the sealant does not impede the motor from turning or interfere with the reassembly of the guiding disk, whirl disk or suction piece (Fig. 1e–g). After drying/curing for 24–48 h, the underside parts should be reassembled onto the motor shaft in opposite order in which they were removed: guiding disk (Figs. 1e, 2j, double arrowheads), whirl disk (Figs. 1f, 2j, single arrowhead) with the suction piece (Figs. 1g, 2j, double arrows) screwed onto the motor shaft firmly to hold the pieces in place (Fig. 2j). The three-piece atomizer ring parts (Figs. 1e; 2j, single arrow) should then be slotted into place on the bottom of the support disk (Fig. 2j). The base’s stand pegs (Fig. 1h) should be reinserted back into their positions. The atomizer is now ready to use.

### Aeroponic chamber

**Fig. 3:**
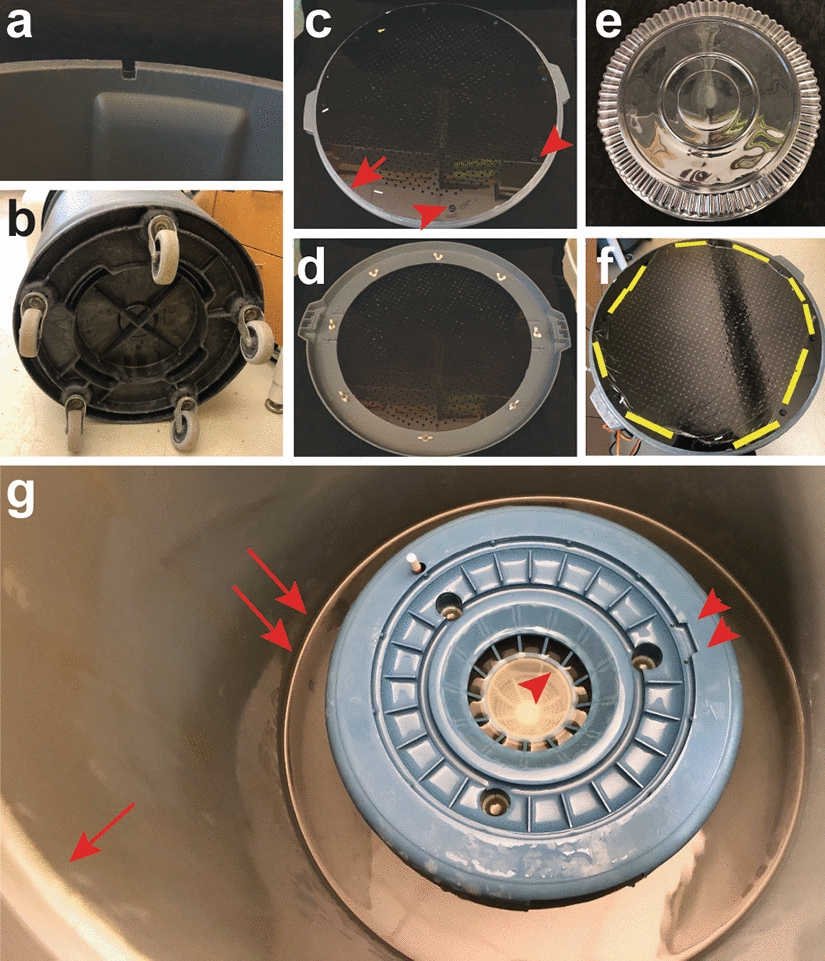
Preparing the aeroponic chamber. **a** A notch is cut into the side of the trash can so that the humidifier’s electric cord can fit in it. **b** Dolly unit attached to the bottom of the trash can. **c** Top view of the trash can lid with the acrylic circle attached to it. Red arrow, silicone sealant adhering to the seam where the disk and trash can top meet. Red arrowheads, two of the screws affixing the acrylic circle in place. **d** Bottom view of the trash can lid with the acrylic circle attached. **e** A plastic catering tray lid is used as a humidity dome. **f** Food service film is stretched across the acrylic circle, cut close to the edges and yellow lab labeling tape is used to secure it to the disk. **g** A disinfecting bleach solution, sterile water for rinses and finally nutrient medium are added sequentially to the trash can (two red arrows) up to the level of the bottom of the base’s integral filter (red arrowhead). The top edge of the trash can is visible (single arrow). The slot in the humidifier base used to orient the “motor” into the base is marked by two arrowheads

The aeroponic chamber is made from a modified gray or dark colored plastic trash can and lid. We use the gray Rubbermaid^©^ Brute 32 gal/121 L trash can. It is modified by cutting a notch in the trash can in which to place the electric cord (Fig. 3a). For convenience in transporting the aeroponic chamber, we use a dolly on which to place the trash can, one that locks onto the trash can (Rubbermaid^©^ Brute dolly) (Fig. 3b).

The lid of the trash can is modified to make a support for the plants as follows. First a hole of 18 inches (46 cm) in diameter is cut in the center of the lid and eight quarter-inch (6.5 mm) holes are drilled around its circumference. Next, a circle of 21 inches (53 cm) diameter, into which 3/16 inch (4 mm) holes that support the plants are drilled at half-inch (12.5 mm) intervals, should be made from a sheet of 3/8 inch (about 9 mm) thick black acrylic sheet using a drill bit designed to cut plastic. Note that these size holes are ideal for the plant we study, *Medicago truncatula*. For other plants with different width roots, different size holes will be appropriate. The acrylic circle should also have holes drilled in it that correspond to the holes drilled into the circumference of the trash can lid to screw it together. The lid and acrylic circle are assembled using plastic screws instead of metal because metal may not be compatible with the nutrient solution that is aerosolized for the plants (Fig. 3c, d). Lastly, the edge of the acrylic circle is sealed with the silicone sealant (Fig. 3c, red arrow). An alternative to the custom acrylic circle is to purchase a sheet of gray polyvinyl chloride (PVC) perforated sheet with straight rows of 1/4" holes on 1/2" centers. This should be cut to fit onto the trash can lid (Additional file 1: Fig. S1) and silicon-sealed.

Investigators should also have available an 18-inch (46 cm) round plastic catering tray lid (Fig. 3e). It is used to cover the aeroponic system top after germinated seeds or plantlets are loaded into the aeroponic chamber to help maintain humidity. Small holes can be made in the catering tray to allow exchange of air. If the plants in the aeroponic system are to be grown in a humidity-controlled chamber, the catering tray lid may not be necessary. As the plants in the aeroponic system grow, they may mature to a stage where the catering tray lid may be propped open to by resting one side on a lab spatula or removed altogether.

### Setting up the aeroponic chamber

The humidifier base should be placed into the trash can and sufficient solution, disinfecting solution, water or nutrient medium, added to bring the liquid up to the level of the top of the base’s integrated filter (Fig. 1h, red arrowhead; Fig. 3g, red arrowheads). The waterproofed, sealed humidifier is then placed on top of the base, matching the slot on the base (Fig. 3g; two red arrowhead) with the notch on the humidifier on the electric cord side. The cord should be brought up through the notch cut into the trash can side. The trash can lid with its integrated acrylic/PVC perforated circle center is then placed onto the trash can. At this point, food service film is placed onto the top of the acrylic/PVC perforated circle over the holes in it and cut to cover its surface. If the food service film isn’t sufficiently wide, it may be necessary to use overlapping pieces. Lab labeling tape can be used to affix the food service film to the edges of the acrylic/PVC perforated circle (Fig. 3f). The food service film over the holes in the acrylic/PVC perforated circle is what supports the plants when they are in the aeroponic system. At this point, the modified humidifier may be turned on. Investigators will notice that the space in between the acrylic/PVC perforated circle and the food service film will become wet after the humidifier is operating. It is a good idea to have a second humidifier sealed and ready as a backup during an experiment, in case there is a leak in the humidifier’s sealant and the resulting electrical short damages the humidifier.

In our hands, the aeroponic chamber may be disinfected, rinsed and run two to five times for 20–25 d experiments before unsealing the humidifier to check its inner parts. The old sealant should be removed by cutting it off and the “motor” inside parts inspected. The humidifier should then be resealed with fresh silicone sealant.

As has been pointed out by David Barker et al. [4], the aeroponic system is not rigorously axenic. Before each use, and again after each use, it is important to disinfect it by running the humidifier in the chamber with commercial household bleach (approximately 5.2–6.2% sodium hypochlorite) diluted 1:10 for at least 30 min and up to several hours. Each time liquid is added to the aeroponic chamber, it should be added up to the level of the bottom of the base’s integral filter (Fig. 3g, red arrowhead). Then the reassembled, waterproofed humidifier motor unit is placed on top of the humidifier base by nesting it into the base using the base’s recessed slot (Fig. 3g, double arrowheads) for orientation. To remove the bleach, remove the humidifier and its base from the trash can and set it aside on a clean surface. The liquid in the trash can may be tipped into a receptacle for further waste treatment. The humidifier base and humidifier are then replaced into the trash can and sterile deionized water should be added and the humidifier left to run for at least 30 min. The process should be repeated at least three to four times until the aeroponic system is thoroughly rinsed, at which point the water should be replaced with the desired nutrient medium, also sterilized. Use care to make sure that liquid levels come up to the bottom of the base’s integral filter with each change. During the sterilization and rinsing process, the transparent food service film should be replaced at each rinse step. The modified trash can lid and plastic catering tray lid may be washed with soap and water, disinfected with 70% ethanol and left to dry in a clean environment. Plant growth experiments should be performed using as clean tools and materials as possible.

### Plant growth method for *Medicago truncatula* nodulation and SNF mutants

After the aeroponic chamber and humidifier are disinfected and rinsed, germinated seeds or plantlets may be loaded into the aeroponic chamber. One of the ways the aeroponic chamber is used is to screen *M. truncatula* mutants for those that have a defect in root nodulation or symbiotic nitrogen fixation (SNF) [10, 11, 15–18]. The following is a method to use the aeroponic chamber for this purpose. The aeroponic system may also be used for experimental growth of plants that have transformed hairy roots [19, 20], in nutrient deprivation studies [5, 21] or other purposes.

Key to success with the aeroponic system is to load surface-sterilized germinated seedlings with radicles of sufficient length so that the developing roots can access the mist aerosolized by the system. Additionally, to use the aeroponic system for screening *M. truncatula* mutants for potential SNF defects, it is important to synchronize *M. truncatula* seed germination with aeroponic system preparation. The humidifier should be sealed, disinfected, well-rinsed and running with nutrient medium in it before the germinated seeds are placed into it. For nodulation studies, actively growing *Sinorhizobium meliloti* cultures are also required several days after the seedlings are added to the aeroponic system.

**Fig. 4:**
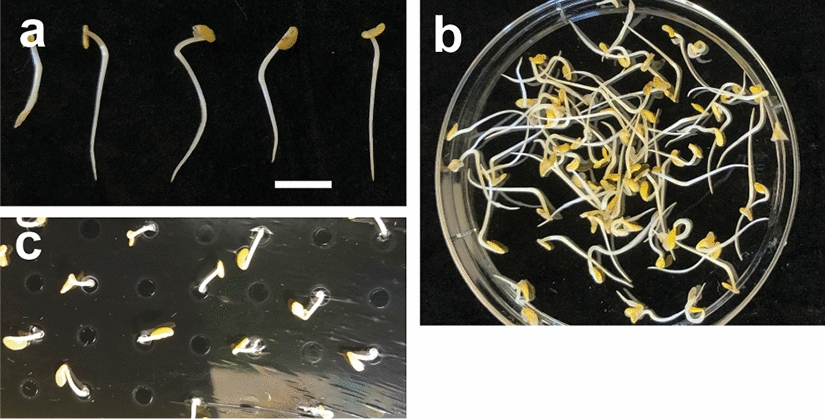
Germinated *M. truncatula* seedlings at the correct stage of growth for the aeroponic system. **a** Seedlings 2 d after being transferred to 1% agarose or Phytoblend for germination. Bar = 1 cm. **b** The seedlings may be agitated gently in sterile water to dislodge their swollen seed coats. **c** Seedling have been placed in an aeroponic chamber through a hole punctured in the food service film. In this example, seedlings were placed every other hole in the acrylic disk on the top of the trash can

As an example, one of our labs used the following procedure to scarify, surface-sterilize and germinate *M. truncatula*; many variations exist. Seeds to be germinated are scarified by agitating them in concentrated sulfuric acid for 6–8 min at RT, followed by five rinses with sterile deionized water at RT. They are then surface-sterilized by shaking them in 6–10% bleach for 90–120 s, followed by five sterilized deionized water rinses at RT. Subsequently, the seeds should be vernalized at 4 °C in the dark for 2–3 d in sterile water. Some investigators vernalize for up to 4–6 d to promote earlier flowering. The sterile water may be changed daily during this time and replaced with 4 °C sterile water. Then, the seeds are transferred to a 1–2% agarose or 1% Phytoblend petri plate and allowed to germinate for 2 d in the dark at RT, upside down so that their emerging radicles point down. At this point the *M. truncatula* seedlings should be up to 3 cm long, depending on the plant (Fig. 4a). The seedlings may be moved to sterilized water for gentle agitation to remove their seed coats (Fig. 4b). Alternatively, the seed coats may be carefully removed with a pointed tip forceps—this latter step may be accomplished as the seedlings are placed in the aeroponic system. Not removing the seed coat will impede the cotyledons from expanding as they grow and could lead to seedling failure. Seedlings that are longer than 1 cm can be moved to the aeroponic chamber (Fig. 4c); shorter ones will not thrive because their root tips will not protrude through the perforated acrylic/PVC circle.

**Fig. 5:**
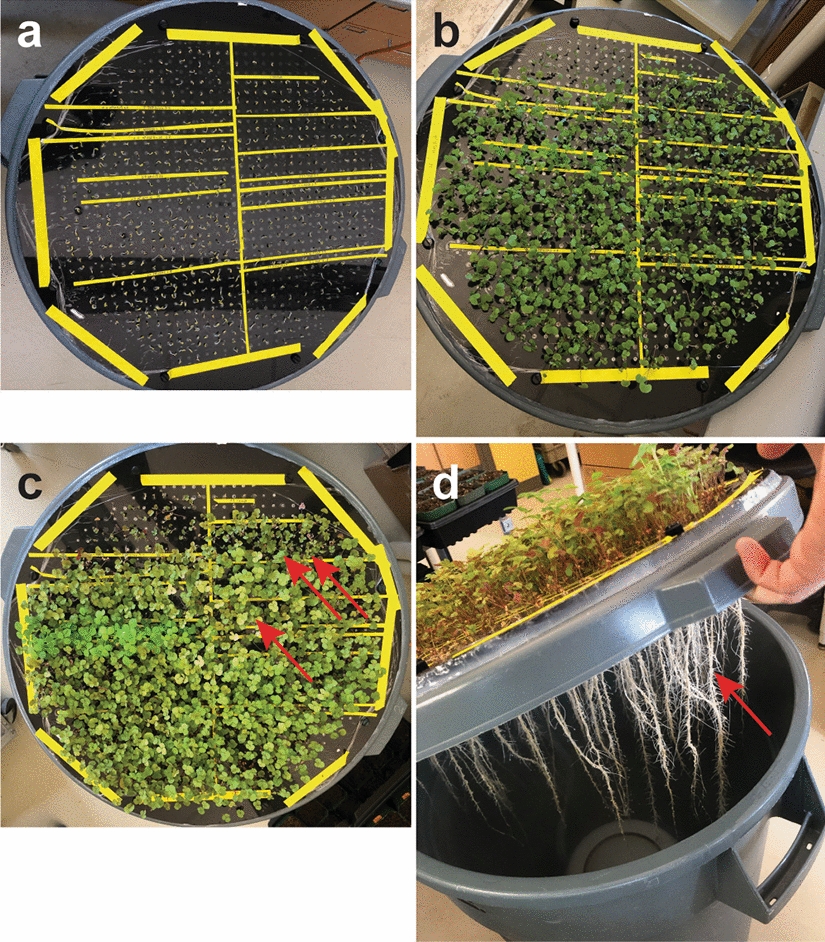
Running the aeroponic system to observe *M. truncatula* symbiotic nitrogen fixation mutants. **a** Sections of the modified trash can lid are demarcated with lab tape. Individual *M. truncatula* seedlings can be seen in each of the sectors, on the day of sowing them into the aeroponic device. **b** *M. truncatula* seedlings 10 days after transfer to the aeroponic system, after 5 d of growth on full N and 5 d in N-starvation medium. **c** *M. truncatula* seedlings 25 d after transfer to the aeroponic system, 15 days post inoculation with *S. meliloti*. Individual mutants within sectors are visible, showing with chlorotic leaves (single arrow). One sector contains a homozygous Fix-genotype demonstrating stunted growth (double arrows). **d** The root system from the aeroponic system in panel c is easy to see after turning off the humidifier unit and lifting the lid of the modified trash can chamber. The arrow points to nodulated roots

We find it convenient to use lab tape to demarcate genotypes of *M. truncatula* seedlings to know their exact locations as we load a chamber (Fig. 5a; Additional file 1: Videos S1 and S2). This is accomplished by cutting lab tape into thin strips as desired and taping it to the food service film covering the top of the aeroponic chamber before beginning to place seed into the chamber. The tape sticks better when the surface is dry. One can use a clean lab wipe to dry the top of the food service film as needed.

Seedlings should be placed into the aeroponic chamber using forceps. It is important to prevent the seedlings from drying out during this process. Sterile water in a clean spray bottle should be sprayed onto the aeroponic chamber surface to maintain humidity. Pointed tip forceps or other sharp instrument should be used to poke the food service film creating a small hole into which a seedling is suspended, with its radicle pointing into the aeroponic chamber and its cotyledons on top. This process is repeated until all the desired seedlings are sown. The distance between each seedling should be determined by how many plants and how long plants are to be grown in the aeroponic chamber. Plants grown close together for a longer period of time may entangle their roots. Seedlings can be planted farther away from each other by skipping holes in the acrylic/PVC circle in the trash can lid (Fig. 4c). During planting, sterile water is sprayed as needed onto the seedlings to continue to keep them moist. Water is also sprayed into the plastic catering tray lid which is used to cover the seedlings to maintain humidity when investigators need a break from loading seedlings onto the aeroponic chamber. The lid should be used for at least the first 5 d of plant growth in the aeroponic chamber.

The nutrient medium we typically use is Lullien’s medium [22]. For screening *M. truncatula* SNF mutants, we grow the plants in Lullien’s medium for 5 d in high N by including 5 mM ammonium nitrate in the medium [17, 18, 20]. The reason for this is to give potential SNF mutants enough of a growth period in full N so they will survive the N starvation that would occur because of their inability to carry out SNF. Other investigators using different growth systems, like cone-tainer systems with Perlite, have used media that contains a low level of bioavailable N, e.g. 2 mM nitrate provided only at the time of planting, to accomplish the N boost [23]. Investigators studying other types of mutants using aeroponic chambers, e.g. autonodulation mutants [10, 24–26], typically skip the high N medium because rhizobia inoculated N-fixing plants will thrive in N starvation. The aeroponic chamber is then wheeled to a growth room and plants allowed to grow at 22 °C on a 16 h/8 h light/dark regime.

At 5 d, the high N medium is discarded and replaced by Lullien’s medium with no N. The seedlings are grown for 5 d on this medium, the medium is again discarded and replaced by the fresh medium of the same composition. By this stage of growth, the plants should show robust growth without apparent signs of N insufficiency (Fig. 5b). After 5 d of no N, 5 mL of a freshly grown, non-saturated *S. meliloti* culture is added to the medium to inoculate the plants for SNF. The culture is first centrifuged at 4000 *g* for 5 min to collect the rhizobia, decanted and resuspended in 5 mL Lullien’s medium with no N. The resuspended *S. meliloti* culture is simply poured into the medium in the aeroponic chamber.

The aeroponic chamber is then placed back into the growth room and plants grown in the same temperature and day/night cycle as before. Plants and medium levels are checked daily. It may be necessary to add sterile water to the aeroponic chamber if the medium level goes half an inch (~ 1.3 cm) below the bottom of the filter unit of the humidifier base (Fig. 3g). Alternatively, the medium could be refreshed every 5 d, depending on the density and age of the plants, or as desired in the particular experiment. To check the levels and the plants, unplug the humidifier and give the chamber 1–3 min for the mist to settle. Set the aeroponic lid off to the side, lift out the humidifier and check the medium level at the humidifier base. Add sterile water as needed to assure that the medium levels stay at the level of the top of the base filter (Fig. 3g). Then replace the humidifier and the lid and restart the humidifier. Plant root growth may be checked at the same time as the nutrient medium level. In wild type *M. truncatula* plants, nascent nodule swellings should become evident at 4–5 days post inoculation (dpi) with rhizobia and be fully emergent at 8–10 dpi (Additional file 1: Fig. S2). After 5 dpi, the humidification chamber (plastic catering tray lid) may be gradually lifted from the top of the aeroponic chamber by placing a clean spatula between it and the aeroponic chamber top at the beginning and progressing to several petri plates to raise the lid up. This will lower the humidity gradually as the plants grow. Alternatively, depending on humidity levels, the plastic catering tray lid may be left in place. We use a non-humidified plant growth room with ambient humidity about 30–50%. By 15 dpi, *M. truncatula* plants that are SNF defective will begin to show signs of chlorosis (Fig. 5c, arrows). The root systems may be visually inspected by turning off the humidifier, allowing the mist to settle and lifting the aeroponic chamber lid (Fig. 5d).

To remove intact plants from the aeroponic chamber, the humidifier is kept running. Using forceps, the holes in the food service film above the chamber’s top are enlarged around an individual plant. The plant is then pulled gently through the hole for phenotyping and further growth if desired, usually by planting into a soil medium. For SNF mutants, it will be important to provide supplemental N in the growth media for growth to seed because the plants will be N starved at this point. If the roots become tangled in the aeroponic chamber, the investigator can gently tease them apart using a gloved hand underneath the lid. If the plants are not desired for further growth, the roots may be cut directly from beneath the aeroponic chamber’s lid. In principle, it is possible to pull plants from the aeroponic chamber and then replace them into the chamber, but once the plants’ roots are longer than 5 cm it is not as easy to orient them properly onto the chamber through holes in the food service film.

A video showing *M. truncatula* seed scarification, germination and placement of seedlings in an aeroponic system is in Additional file 1: Video S1. A second video showing a running aeroponic system with *M. truncatula* seedlings is in Additional file 1: Video S2.

### Customization for other uses

One of the original aeroponic chamber designs made use of larger holes in the top, large enough to accommodate 1.8 mL Eppendorf-type tubes filled with nutrient media solidified in 0.8% agarose with their bottoms cut off. *M. truncatula* seeds were germinated in the 1.8 mL tubes with the tubes suspended over a nutrient solution. Once the seeds germinated and their radicles protruded from the end of the cut tube, the tube was pressed into the aeroponic chamber lid. Similar use can be made of small PCR type tubes or pipette tips with their bottoms cut off. Either aeroponic top system, tube or food service film, could be adapted for plants with different sized root systems. With the tube system, once the plants are grown to the desired endpoint, the entire tube may be lifted from the aeroponic system lid. Or if the root system is too large, it may be cut off beneath the lid. Examples where investigators included plants grown this way include two large scale gene expression studies [27, 28]. For larger plants like pea, bean or soybean, the holes that are drilled in the acrylic/PVC perforated circle may need to be larger to accommodate these plants’ thicker roots being placed directly into the aeroponic chamber. Alternately, researchers could germinate seeds in rockwool and place the rockwool plugs in the holes in the aeroponic system top.

We routinely use the aeroponic system for growing and studying composite plants with *Agrobacterium rhizogenes* transformed hairy roots [19, 20]. After identifying transformed roots and trimming away those root sections that are not transformed, the composite plants’ roots are gently placed into the aeroponic system lid. It may be necessary to tear away all the food service film from around the aeroponic system lid individual hole when accomplishing this. After the composite plant is in the lid’s hole, thin strips of food service film may be placed around it, assuring that it will stay in place until the composite plant grows larger. This technique was used in a plant complementation assay to assess functional implications of structural changes to a nutrient transporter essential for nodulation [20].

This system can be considered part of a medium throughput phenotyping system. Plants can be removed from the chamber, photographed and analyzed by a program like ImageJ [29] or Fiji [30]. An example of a phenotyping experiment, with images of the plants and their roots in Additional file 1: Fig. S2 and quantitative data in Additional file 1: Fig. S3. Once the plants are examined for phenotypes, they can be easily transferred to soil for additional growth. Another example is shown in Additional file 1: Fig. S4, where plants were allowed to grow longer root systems in full N conditions for 11 d, then N starved and inoculated with *S. meliloti* for a nodule developmental series, similar to experiments reported [27, 28].

## Conclusions

A convenient growth system is essential for researchers to investigate plant root systems for many different types of study. Here we described an aeroponic system originally intended for nodulating legume root systems that was designed by a French engineer and modified by members of the legume community including us. This system presents many advantages to researchers studying root growth and development. Investigators can grow hundreds of plants at one time with this aeroponic system. Although not a sterile system, it can be kept axenic through careful laboratory practice. The nutrient medium can be altered during plant growth depending on experimental needs. Researchers can easily remove plants from the aeroponic chamber without cleaning particulates or soil from the root systems, making it possible to easily visualize the root system for phenotyping. With care, it is easy to remove plants from the aeroponic system without damaging the root systems.

The growth chamber of our modified aeroponic system is deep enough to grow *M. truncatula*, which have long roots. Although our typical growth period is 25 days, plants may be grown for longer periods of time, if desired.

There are some drawbacks to using an aeroponic chamber. Although mixed inoculation with different rhizobia species are possible with the aeroponic system, a drawback to using this system where it is desired to compare legume responses to different rhizobia is the need to have different aeroponic systems for each isolated rhizobium strain. Another disadvantage is that the rhizobia are aerosolized, and thus, the rhizobia may easily contaminate the growth facility where the aeroponic chamber is placed. This may necessitate having separate facilities that are rhizobia-free when growth of legumes without rhizobia is desired. The aerosolized rhizobia may also present issues with bio-containment and biosafety protocols, although rhizobia are not known pathogens. Caveats of using aeroponic systems include the likelihood that root growth in aeroponic systems produces roots having physiological and biochemical differences from those grown on soil or other solid media. For example, it may be expected that roots will have continual depletion of water-soluble exudates from them. We have previously speculated that this may explain differences seen in organic acid contents of root and nodules in phosphate deprivation observed in between plants grown aeroponically versus those grown on a solid support [5]. These plant exudates could normally accumulate nonuniformly in specific root zones, potentially conditioning micro-environments affecting root physiology or root microbiomes, rendering the aeroponic system less useful for these types of studies. Plant roots in aeroponic systems may develop mechanically weaker cell walls than those grown on solid supports, because they do not have to push their way through the support. These could induce specific stress responses. The mechanostimulation of the misting in an aeroponic system could alter stress responses in roots [31]. Plants removed from the aeroponic system that are subsequently potted on soil may need humid conditions for a few days to acclimate to the soil. In addition, plant species that thrive in deserts or other dry environments may be unsuitable for study in aeroponic systems.

## Methods

*S. meliloti* strain 1021 was obtained from Fred Ausubel and was originally named *Rhizobium meliloti* 1021 [32]. *M. truncatula* wild type R108 and *Tnt1* mutants in the R108 genetic background [23, 33] were obtained from Jiangqi Wen, via the *Tnt1* mutant collection database. See https://medicago-mutant.dasnr.okstate.edu/mutant/index.php.

Lullien’s medium was used as described [22].

The sources and approximate costs of materials to build an aeroponic system are given in Table 1.

**Table 1:** Component sources and component for an aeroponic chamber

Component, brand used	Size	Approximate cost per unit (USD)	Supplier
Humidifier, Condair 505	Each	$1200	Condair Ltd, Pfäffikon, Switzerland; Texas AirSystems, Irving, TX, USA
ASI Aquarium Sealant	10.2 oz tube, each	$18	Best Materials, Phoenix, AZ, USA
Trash can, with lid; Rubbermaid Brute	32 gal can, each	$40	Home Depot, Denton, TX, USA
Trash Can Dolly, Rubbermaid Brute	5 wheels, each	$43	Home Depot, Denton, TX, USA
Acrylic black opaque sheet	36″ × 48″ × 3/8″, each; one sheet makes four tops	$250	Home Depot, Denton, TX, USA
Polyvinyl chloride (PVC) gray perforated sheet, (alternative to acrylic sheet)	1/4" × 24" × 48" perforated sheet with straight rows of 1/4" holes on 1/2" centers; other perforation densities available; one sheet makes two tops	$79	United States Plastic Corp., Lima, OH, USA
Plastic screws Cheese head bolts, nylon	1/4″ wide, package/20	$10	Amazon, USA
Nuts, nylon	Package/30	$11	Amazon, USA
Food service film	18″ × 3000’, roll	$35	Amazon, USA
Round clear plastic catering tray high dome lid	18″ wide, package/5	$9	Webstaurant Store, USA
Total cost		$1616 or $1445 Depends on type of plant support lid (acrylic or PVC sheet)	

## Supplementary Information

**Additional file 1: File S1.** Examples where investigators used an aeroponic system identical or similar to the one described; selected publications, with studies in *M. truncatula*, *Lotus japonicus*, alfalfa (*M. sativa*) and pea (*Pisum sativum*). **Figure S1.** Alternative pre-perforated PVC aeroponic system top. **Figure S2.** Phenotype of plants and nodules during SNF. **Figure S3.** Growth of plants and nodules during SNF. **Figure S4.** Longer growth of plant and nodules during SNF. **Video S1.** Growing *M. truncatula* for screening a mapping population. **Video S2.** *M. truncatula* growing in a running aeroponic system.

## Data Availability

Not applicable.
