# Correction: A modified aeroponic system for growing small-seeded legumes and other plants to study root systems

**DOI:** 10.1186/s13007-023-01007-z

**Published:** 2023-03-25

**Authors:** Jingya Cai, Vijaykumar Veerappan, Kate Arildsen, Catrina Sullivan, Megan Piechowicz, Julia Frugoli, Rebecca Dickstein

**Affiliations:** 1 Department of Biological Sciences and BioDiscovery Institute, University of North Texas, Denton, TX 76203 USA; 2 Department of Biology, Eastern Connecticut State University, Willimantic, CT 06226 USA; 3 Department of Genetics and Biochemistry, Clemson University, Clemson, SC 29634 USA

**Correction: Plant Methods (2023) 19:21** 10.1186/s13007-023-01000-6

In the original publication of the article [1], the affiliation of the first author was published incorrectly. Hence, the order of affiliations was renumbered and the affiliation of the first author was corrected. The corrected affiliations of the authors is given in this Correction article. The original article has been corrected.

## References

[1] Cai J, Veerappan V, Arildsen K, Sullivan C, Piechowicz M, Frugoli J, Dickstein R (2023). A modified aeroponic system for growing small-seeded legumes and other plants to study root systems. Plant Methods.

