# Supplementary material for: A modified aeroponic system for growing small-seeded legumes and other plants to study root systems
Source: Plant Methods. 2023 Mar 3;19:21. doi: 10.1186/s13007-023-01000-6 (PMC9983192; doi:10.1186/s13007-023-01000-6)
Supplement: Supplementary file 1 — Additional file 1: File S1. Examples where investigators used an aeroponic system identical or similar to the one described; selected publications, with studies in M. truncatula, Lotus japonicus, alfalfa (M. sativa) and pea (Pisum sativum). Figure S1. Alternative pre-perforated PVC aeroponic system top. Figure S2. Phenotype of plants and nodules during SNF. Figure S3. Growth of plants and nodules during SNF. Figure S4. Longer growth of plant and nodules during SNF. Video S1. Growing M. truncatula for screening a mapping population. Video S2. M. truncatula growing in a running aeroponic system. [file 13007_2023_1000_MOESM1_ESM.pdf]

Cai et al. Additional file for **A Modified Aeroponic System for Growing Small-Seeded Legumes and Other Plants to Study Root Systems.** https://doi.org/10.1186/s13007-023-01000-6.

File S1: Examples where investigators used an aeroponic system identical or similar to the one described; selected publications, with studies in *M. truncatula*, *Lotus japonicus*, alfalfa (*M. sativa*) and pea (*Pisum sativum*).

Medicago truncatula

Barker DG, Pfaff T, Moreau D, Groves E, Ruffel S, Lepetit M, Whitehand S, Maillet F, Nair RM, Journet E-P: **Growing *M. truncatula:* choice of substrates and growth conditions** In: *The Medicago truncatula handbook.* Edited by Mathesius U, Journet E-P, Sumner LW. http://www.noble.org/MedicagoHandbook/: ISBN 0-9754303-1-9; 2006.

Catalano C, Lane WS, Sherrier DJ: **Biochemical characterization of symbiosome membrane proteins from *Medicago truncatula* root nodules.** *Electrophoresis* 2004, **25**:519-531.

Dokwal D, Romsdahl TB, Kunz DA, Alonso AP, Dickstein R: **Phosphorus deprivation affects composition and spatial distribution of membrane lipids in legume nodules**. *Plant Physiol* 2021, **185**:1847-1859.

Dokwal D, Cocuron J-C, Alonso AP, Dickstein R: **Metabolite shift in *Medicago truncatula* occurs in phosphorus deprivation.** *J Exp Bot* 2022, **73**:2093–2111.

Dreher D, Baldermann S, Schreiner M, Hause B: **An arbuscular mycorrhizal fungus and a root pathogen induce different volatiles emitted by *Medicago truncatula* roots**. *Journal of Advanced Research* 2019, **19**:85-90.

Durgo H, Klement E, Hunyadi-Gulyas E, Szucs A, Kereszt A, Medzihradszky KF, Kondorosi E: **Identification of nodule-specific cysteine-rich plant peptides in endosymbiotic bacteria**. *Proteomics* 2015, **15**:2291-2295.

Gao Y, Selee B, Schnabel EL, Poehlman WL, Chavan SA, Frugoli JA, Feltus FA: **Time series transcriptome analysis in *Medicago truncatula* shoot and root tissue during early nodulation**. *Frontiers in Plant Science* 2022, **13**:861639

Gaudioso-Pedraza R, Beck M, Frances L, Kirk P, Ripodas C, Niebel A, Oldroyd GED, Benitez-Alfonso Y, de Carvalho-Niebel F: **Callose-regulated symplastic communication coordinates symbiotic root nodule development**. *Curr Biol* 2018, **28**:3562-3577.

Jardinaud M-F, Carrere S, Gourion B, Gamas P: **Symbiotic nodule development and efficiency in the *Medicago truncatula Mtefd-1* mutant is highly dependent on *Sinorhizobium* strains**. *Plant & Cell Physiology* 2022:pcac134

Jiang S, Jardinaud M-F, Gao J, Pecrix Y, Wen J, Mysore K, Xu P, Sanchez-Canizares C, Ruan Y, Li Q, Zhu M, Li F, Wang E, Poole PS, Gamas P, Murray JD: **NIN-like protein transcription factors regulate leghemoglobin genes in legume nodules**. *Science* 2021, **374**:625-628.

Kim G-B, Son S-U, Yu H-J, Mun J-H: ***MtGA2ox10* encoding C20-GA2-oxidase regulates rhizobial infection and nodule development in *Medicago truncatula***. *Sci Rep* 2019, **9**:5952.

Larrainzar E, Riely BK, Kim SC, Carrasquilla-Garcia N, Yu H-J, Hwang H-J, Oh M, Kim GB, Surendrarao AK, Chasman D, Siahpirani AF, Penmetsa RV, Lee G-S, Kim N, Roy S, Mun J-H, Cook DR: **Deep sequencing of the *Medicago truncatula* root transcriptome reveals a massive and early interaction between Nodulation factor and ethylene signals**. *Plant Physiol* 2015, **169**:233-265.

Meng J, Peng M, Yang J, Zhao Y, Hu J, Zhu Y, He H: **Genome-wide analysis of the cyclin gene family and their expression profile in *Medicago truncatula***. *International Journal of Molecular Sciences* 2020, **21**:9430.

Meng J, Yang J, Peng M, Liu X, He H: **Genome-wide characterization, evolution, and expression analysis of the leucine-rich repeat receptor-like protein kinase (LRR-RLK) gene family in *Medicago truncatula*** *Life (Basel)* 2020, **10**:176.

Pecrix Y, Sallet E, Moreau S, Bouchez O, Carrere S, Gouzy J, Jardinaud M-F, Gamas P: **DNA demethylation and hypermethylation are both required for late nodule development in *Medicago***. *Nat Plants* 2022, **8**:741-749.

Penmetsa RV, Cook DR: **A legume ethylene-insensitive mutant hyperinfected by Its rhizobial symbiont**. *Science* 1997, **275**:527-530.

Penmetsa RV, Cook DR: **Production and characterization of diverse developmental mutants of *Medicago truncatula***. *Plant Physiol* 2000, **123**(4):1387-1398.

Penmetsa RV, Frugoli JA, Smith LS, Long SR, Cook DR: **Dual genetic pathways controlling nodule number in *Medicago truncatula***. *Plant Physiol* 2003, **131**(3):998-1008.

Poehlman W, Schnabel E, Chavan S, Frugoli J, Feltus FA: **Identifying temporally regulated root nodulation biomarkers using time series gene co-expression network analysis**. *Frontiers in Plant Science* 2019, **10**:1409.

Schnabel E, Journet E-P, de Carvalho-Niebel F, Duc G, Frugoli J: **The *Medicago truncatula SUNN* gene encodes a *CLV1*-like leucine-rich repeat receptor kinase that regulates nodule number and root length**. *Plant Mol Biol* 2005, **58**:809–822.

Schnabel E, Mukherjee A, Smith L, Kassaw T, Long S, Frugoli J: **The *lss* supernodulation mutant of *Medicago truncatula* reduces expression of the *SUNN* gene**. *Plant Physiol* 2010, **154**:1390-1402.

Schnabel EL, Kassaw TK, Smith LS, Marsh JF, Oldroyd GED, Long SR, Frugoli JA: **The *ROOT DETERMINED NODULATION1* gene regulates nodule number in roots of *Medicago truncatula* and defines a highly conserved, uncharacterized plant gene family**. *Plant Physiol* 2011, **157**:328-340

Schnabel E, Chavan S, Gao Y, Poehlman WL, Feltus FA, Frugoli J: **Transcriptome analysis of *Medicago truncatula* Autoregulation of Nodulation mutants reveals that disruption of the SUNN pathway causes constitutive expression changes in a small group of genes, but the overall response to rhizobia resembles wild type, including induction of *TML1* and *TML2***. *bioRxvi doi: 101101/20230119524769* 2023.

Veereshlingam H, Haynes JG, Sherrier DJ, Penmetsa RV, Cook DR, Dickstein R: ***nip*, a symbiotic *Medicago truncatula* mutant that forms root nodules with aberrant infection threads and plant defense-like response**. *Plant Physiol* 2004, **136**:3692-3702.

Yadav H, Dreher D, Athmer B, Porzel A, Gavrin A, Baldermann S, Tissier A, Hausea B: **Medicago TERPENE SYNTHASE 10 Is Involved in Defense Against an Oomycete Root Pathogen**. *Plant Physiol* 2019, **180**:1598-1613.

Yu Y-C, Dickstein R, Longo A: **Structural modeling and *in planta* complementation studies link mutated residues of the *Medicago truncatula* nitrate transporter NPF1.7 to functionality in root nodules**. *Frontiers in Plant Science* 2021, **12**:685334.

Lotus japonicus

Henzler T, Waterhouse RN, Smyth AJ, Carvajal M, Cooke DT, Schäffner AR, Steudle E, Clarkson DT: **Diurnal variations in hydraulic conductivity and root pressure can be correlated with the expression of putative aquaporins in the roots of *Lotus japonicus***. *Planta* 1999, **210**:50–60.

Zdyb A, Salgado MG, Demchenko KN, Brenner WG, Płaszczyca M, Stumpe M, Herrfurth C, Feussner I, Pawlowski K: **Allene oxide synthase, allene oxide cyclase and jasmonic acid levels in *Lotus japonicus* nodules.** *PLoS One* 2018, **138**:e0190884.

Alfalfa (*M. sativa*)

Lullien V, Barker DG, de Lajudie P, Huguet T: **Plant gene expression in effective and ineffective root nodules of alfalfa (*Medicago sativa*).** *Plant Mol Biol* 1987, **9**:469-478.

Pea (*Pisum sativum*)

Lebedeva MA, Sadikova DS, Dobychkina D, Zhukov VA, Lutova LA: ***CLAVATA3/EMBRYO SURROUNDING REGION* genes involved in symbiotic nodulation in *Pisum sativum*** *Agronomy* 2022, **12**:2840.

Vedam V, Kannenberg E, Datta A, Brown D, Haynes-Gann JG, Sherrier DJ, Carlson RW: **The pea nodule environment restores the ability of a *Rhizobium leguminosarum* lipopolysaccharide *acpXL* mutant to add 27-hydroxyoctacosanoic acid to its lipid A**. *J Bacteriol* 2006, **188**:2126-2133.

Cai et al. Additional file for **A Modified Aeroponic System for Growing Small-Seeded Legumes and Other Plants to Study Root Systems.** https://doi.org/10.1186/s13007-023-01000-6.

Fig. S1

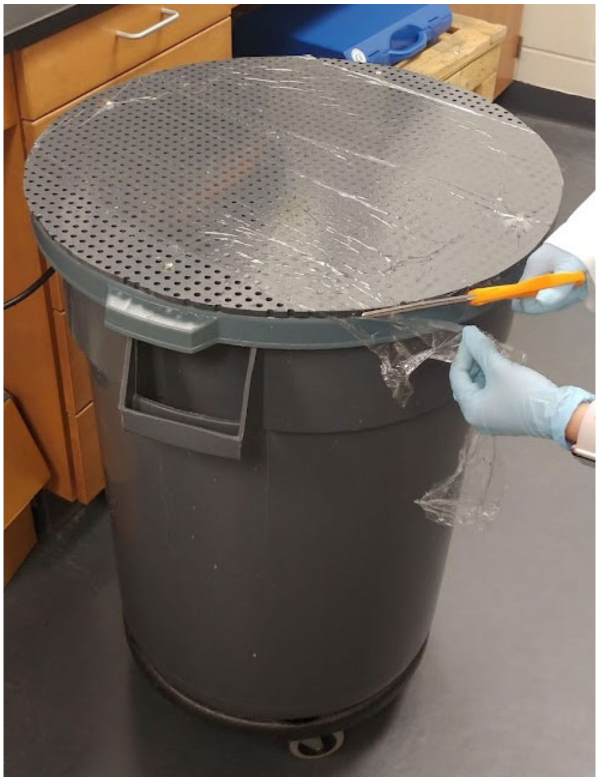

Fig. S1. Alternative pre-perforated PVC aeroponic system top. An alternative to drilling holes in an acrylic sheet is fitting a perforated PVC sheet to the trash can lid to fabricate an aeroponic system top. Here food service film is stretched tight over a sanitized lid with the excess trimmed off. The food service film must cover all of the holes on the top to the edge of the large hole cut into the trash can lid.

Cai et al. Additional file for **A Modified Aeroponic System for Growing Small-Seeded Legumes and Other Plants to Study Root Systems.** https://doi.org/10.1186/s13007-023-01000-6.

Fig. S2

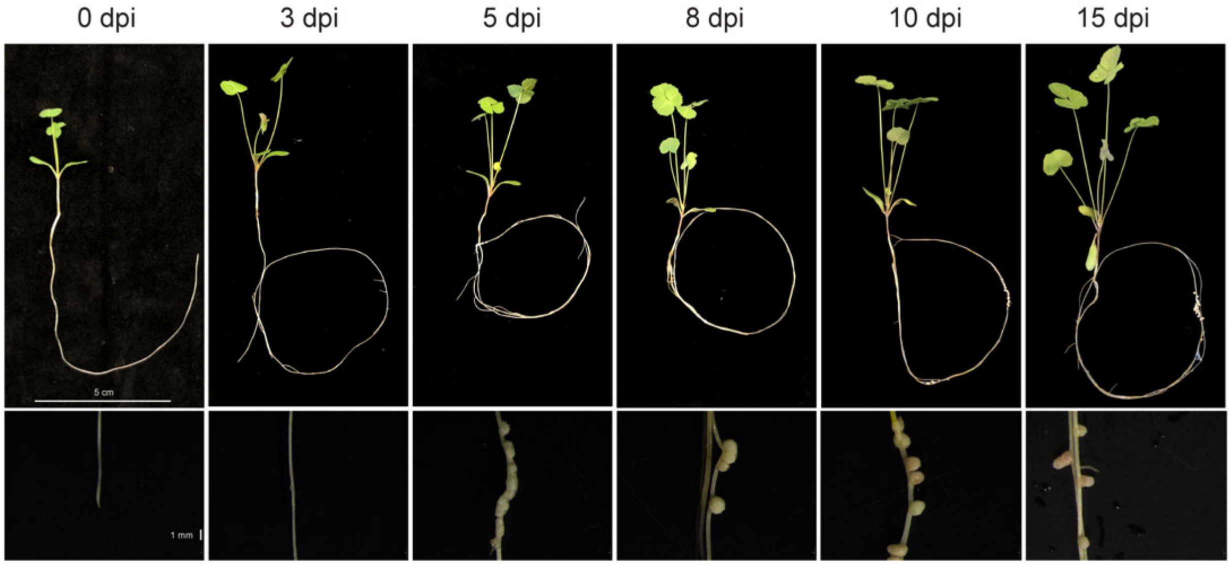

Fig. S2. Phenotype of plants and nodules during SNF.
Wild-type *M. truncatula* R108 plants were grown in an aeroponic system on full nitrogen (N) in Lullien's medium for 5 d, starved for 5 d and inoculated with *Sinorhizobium meliloti* strain *Sm1021*. Plants (top) and nodulated roots (bottom) were observed at 0 days post inoculation (dpi), 3 dpi, 5 dpi, 8 dpi, 10 dpi and 15 dpi.

Cai et al. Additional file for **A Modified Aeroponic System for Growing Small-Seeded Legumes and Other Plants to Study Root Systems.** https://doi.org/10.1186/s13007-023-01000-6.

Fig. S3

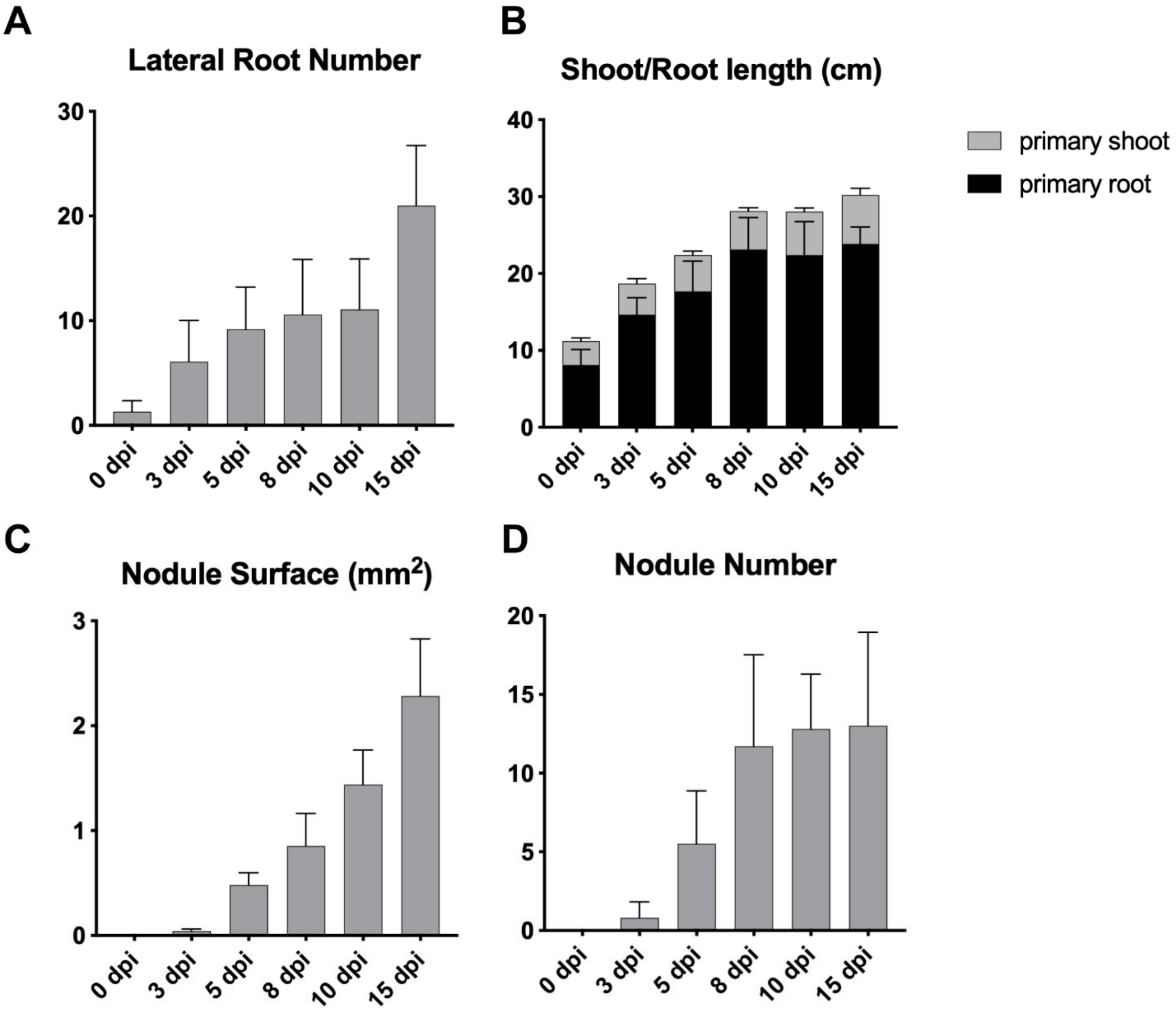

Fig. S3. Growth of plants and nodules during SNF.
Wild-type *M. truncatula* R108 plants were grown in an aeroponic system on full nitrogen (N) in Lullien's medium for 5 d, starved for 5 d and inoculated with *Sinorhizobium meliloti* strain *Sm1021*. Plants and nodulated roots were observed at 0 days post inoculation (dpi), 3 dpi, 5 dpi, 8 dpi, 10 dpi and 15 dpi. N=10. (A) Lateral roots were enumerated. N=10. (B) Shoot and root length were measured. (C) Nodule surface was measured from nodule images using Fiji software. N=10. (D) Nodules were observed and counted. N=10.

Reference for Fiji software:

Schindelin J, Arganda-Carreras I, Frise E, Kaynig V, Longair M, Pietzsch T, Preibisch S, Rueden C, Saalfeld S, Schmid B, Tinevez J-Y, White DJ, Hartenstein V, Eliceiri K, Tomancak P, Cardona A: **Fiji: an open-source platform for biological-image analysis**. *Nature Methods* 2012, **9**(9):676-682

Cai et al. Additional file for **A Modified Aeroponic System for Growing Small-Seeded Legumes and Other Plants to Study Root Systems.** https://doi.org/10.1186/s13007-023-01000-6.

Fig. S4

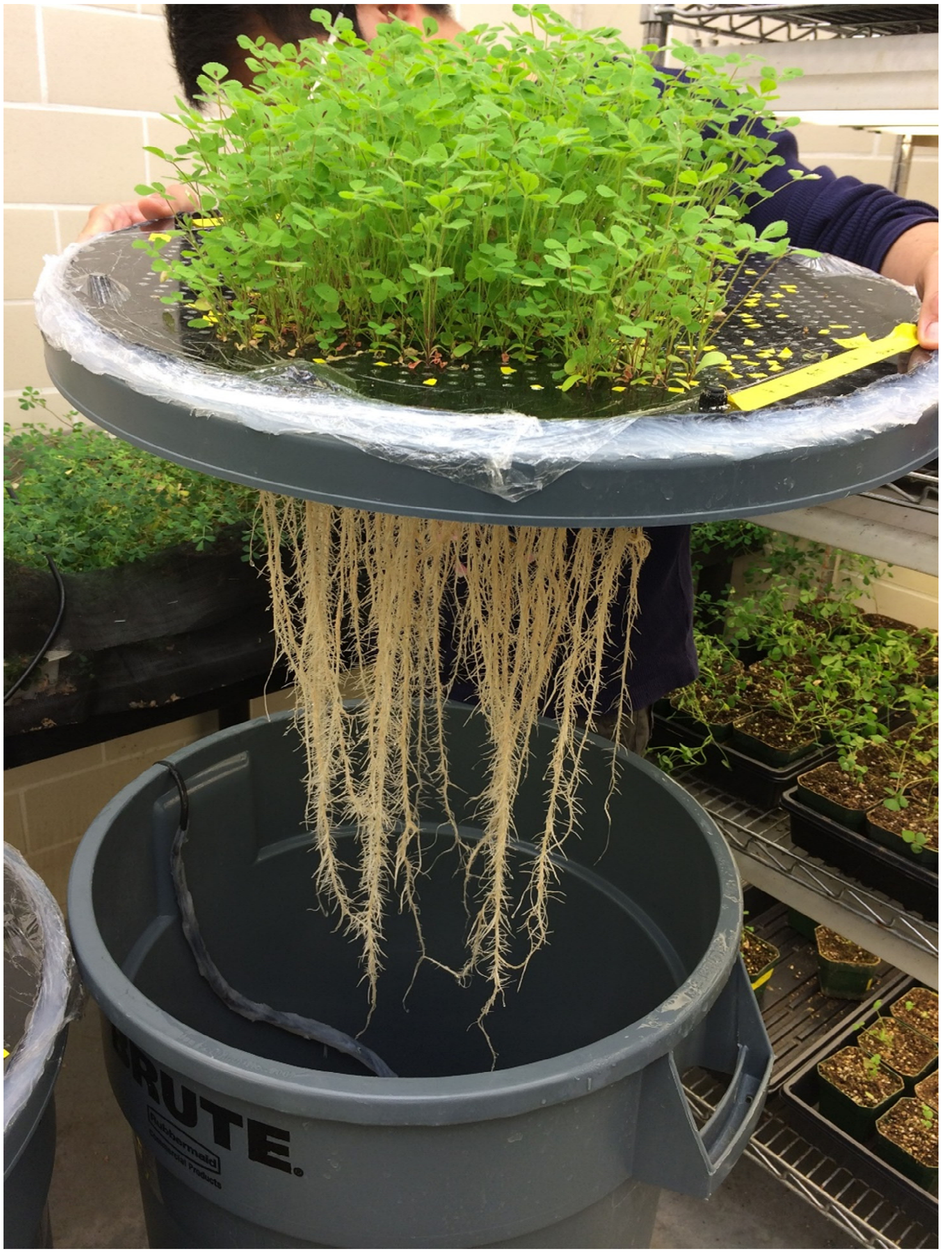

Fig. S4. Longer growth of plant and nodules during SNF.
Wild-type *M. truncatula* A17 were grown in an aeroponic system on full nitrogen (N) in Lullien's medium for 11 d, starved for 5 d and inoculated with *Sinorhizobium meliloti* strain *Sm1021*. Plants with nodulated roots were observed at approximately ten days post inoculation.

Cai et al. Additional file for **A Modified Aeroponic System for Growing Small-Seeded Legumes and Other Plants to Study Root Systems.** **https://doi.org/10.1186/s13007-023-01000-6**.

Video S1. Growing *M. truncatula* for screening a mapping population. This video shows how to harvest seed from *M. truncatula* pods, the steps of scarifying and germinating seeds, and, starting at 4:12, loading germinated seedlings onto a prepared aeroponic system (caisson) lid. https://youtu.be/AbRd9gB2IaA.

Video S2. *M. truncatula* growing in a running aeroponic system. This video shows an aeroponic chamber several days after germinated seedlings have been loaded onto the lid. For observation of the growing plants, it is usually recommended to open the lid after the aeroponic system motor has been turned off for several minutes to give the mist time to settle; not as shown in the video. https://youtu.be/7F1-TTfTLgc.
